# Supplementary material for: Design and Development of Tools for Risk Evaluation of Diabetes and Cardiovascular Disease in Community Pharmacy
Source: Int J Environ Res Public Health. 2023 Feb 5;20(4):2819. doi: 10.3390/ijerph20042819 (PMC9956138; doi:10.3390/ijerph20042819)
Supplement: Supplementary file 1 [file ijerph-20-02819-s001.zip › Questionnaire A - Evaluation of the lifestyle booklet.pdf]

Questionnaire A-
Evaluation of the patient booklet

Participant n°……..

Participant's characteristics

☐ Man ☐ Woman ☐ Other

Age:

- ☐ 25 – 35 years old
- ☐ 36 – 45 years old
- ☐ 46 – 55 years old
- ☐ 56 – 65 years old

Education level:

- ☐ No education or primary education
- ☐ High school
- ☐ Bachelor's degree
- ☐ Master's degree

PART 1 - Understanding

1. **After reading the booklet, can you tell me in your own words what it is about?**

   ☐ Yes ☐ Nope ☐ Hardly

   Comment:

2. **Do you find information in the booklet on how to eat healthier?**

   ☐ Yes ☐ Nope ☐ Hardly

   Comment:

3. **Can you give me some examples of how to eat more balanced?**

   The answer indicates an understanding of the text

   ☐ Yes ☐ Nope ☐ Hardly

   Comment:

4. **Do you find information in the booklet on how to be more active?**

   ☐ Yes ☐ Nope ☐ Hardly

   Comment:

5. **Can you give me some examples on how to move more?**

   The answer indicates an understanding of the text

   ☐ Yes ☐ Nope ☐ Hardly

Comment:

6. **Do you find information in the booklet on how to reduce stress?**

☐ Yes ☐ Nope ☐ Hardly

Comment:

7. **Can you give me some examples of how to reduce stress?**

The answer indicates an understanding of the text

☐ Yes ☐ Nope ☐ Hardly

Comment:

PART 2 – Perceptions

How would you qualify the following statements?

Objectives

1. **At first glance, this booklet attracted my attention**

☐ Strongly agree ☐ Agree ☐ Neither agree, nor disagree ☐ Disagree ☐ Strongly disagree

How could this booklet be made more attractive to you?

Comment:

2. **It held my attention**

☐ Strongly agree ☐ Agree ☐ Neither agree, nor disagree ☐ Disagree ☐ Strongly disagree

Why? How could this booklet be made more interesting for you?

Comment:

3. **It is useful**

☐ Strongly agree	☐ Agree	☐ Neither agree, nor disagree	☐ Disagree	☐ Strongly disagree

Why? How could this booklet be made more useful to you?

Comment:

4. **I will recommend it to a friend or relative to read**

☐ Strongly agree	☐ Agree	☐ Neither agree, nor disagree	☐ Disagree	☐ Strongly disagree

Could you tell me why?

Comment:

Content and topics

5. **I believe what is written**

☐ Strongly agree ☐ Agree ☐ Neither agree, nor disagree ☐ Disagree ☐ Strongly disagree

Is there a part that you find less believable? Could you tell me why?

Comment:

6. **What it says is important**

☐ Strongly agree ☐ Agree ☐ Neither agree, nor disagree ☐ Disagree ☐ Strongly disagree

Why ? Is there a specific part that you think is less important?

Comment:

7. **It reminds me of some things I need to think about**

☐ Strongly agree	☐ Agree	☐ Neither agree, nor disagree	☐ Disagree	☐ Strongly disagree

Could you give me some examples? If not, are there things we could add that would be more helpful to you?

Comment:

8. **It gives me new ideas or leads to implement**

☐ Strongly agree	☐ Agree	☐ Neither agree, nor disagree	☐ Disagree	☐ Strongly disagree

Are these things you already know? On what subject would you like us to add new ideas/news?

Comment:

9. It breaks down the advice into achievable steps for me

☐ Strongly agree	☐ Agree	☐ Neither agree, nor disagree	☐ Disagree	☐ Strongly disagree

What advice doesn't seem reachable to you? Why? How could we make them more concrete?

Comments:

10. It changes some of my thinking

☐ Strongly agree	☐ Agree	☐ Neither agree, nor disagree	☐ Disagree	☐ Strongly disagree

Why? Could you give me some examples?

Comment:

11. It could change how I do things

☐ Strongly agree	☐ Agree	☐ Neither agree, nor disagree	☐ Disagree	☐ Strongly disagree

Why? Could you give me some examples?

Comment:

Layout

12. It is easy to read

☐ Strongly agree ☐ Agree ☐ Neither agree, nor disagree ☐ Disagree ☐ Strongly disagree

Why? How could we make the booklet easier for you to read?

Comment:

13. It is easy to understand

☐ Strongly agree ☐ Agree ☐ Neither agree, ☐ Disagree ☐ Strongly disagree

nor disagree

Were there any words that were difficult for you to understand? How could we make the booklet easier to understand for you?

Comment:

14. The different sections make the document easy to navigate

☐ Strongly agree	☐ Agree	☐ Neither agree, nor disagree	☐ Disagree	☐ Strongly disagree

Why? How could we make the booklet easier to navigate for you?
Could tabs for browsing the booklet help you?

Comment:

15. I like illustrations

☐ Strongly agree	☐ Agree	☐ Neither agree, nor disagree	☐ Disagree	☐ Strongly disagree

Why? Do you think the photos represent you?

Comment:

16. The illustrations are easy to understand

☐ Strongly agree	☐ Agree	☐ Neither agree, nor disagree	☐ Disagree	☐ Strongly disagree

Why? What makes it difficult to understand? Could you show me?

Comment:

Conclusion

17. What do you think of the "28 days to improve myself" part?
Personally, would you use this part? Why ?

18. In general, what do you think of the booklet and the information it contains?
What are the positive points? What are the negative points?

Comment:

19. **Are there any sections/information you would like to have that are missing from the booklet?**

Comment:

20. **Is there anything else we haven't covered that you would like to mention?**

Comment:
