# Supplementary material for: Design and Development of Tools for Risk Evaluation of Diabetes and Cardiovascular Disease in Community Pharmacy
Source: Int J Environ Res Public Health. 2023 Feb 5;20(4):2819. doi: 10.3390/ijerph20042819 (PMC9956138; doi:10.3390/ijerph20042819)
Supplement: Supplementary file 1 [file ijerph-20-02819-s001.zip › Questionnaire B - Evaluation of the folders.pdf]

Questionnaire B –
Evaluation of the folders

BROCHURE: diabetes / cardiovascular diseases

Participant n°……..

Participant's characteristics

☐ Man ☐ Woman ☐ Other

Age:

- ☐ 25 – 35 years old
- ☐ 36 – 45 years old
- ☐ 46 – 55 years old
- ☐ 56 – 65 years old

Education level:

- ☐ No education or primary education
- ☐ High school
- ☐ Bachelor's degree
- ☐ Master's degree

PART 1 - Understanding

1. After reading the folder, can you tell me in your own words what it is about?

☐ Yes ☐ Nope ☐ Hardly

Comments:

2. Do you find explanations on what diabetes/cardiovascular disease is?

☐ Yes ☐ Nope ☐ Hardly

Comments:

3. Can you tell me what you understood about diabetes/cardiovascular disease?

The answer indicates an understanding of the text

☐ Yes ☐ Nope ☐ Hardly

Comments:

4. Does the folder explain what puts a person at risk for diabetes/cardiovascular disease?

☐ Yes ☐ Nope ☐ Hardly

Comments:

5. **Can you tell me what puts a person at risk for diabetes/cardiovascular disease?**
   The answer indicates an understanding of the text
   ☐ Yes ☐ Nope ☐ Hardly

   Comments:

6. **Does the folder give advice on how to avoid diabetes/cardiovascular disease?**
   Can you find some of them in the brochure?
   ☐ Yes ☐ Nope ☐ Hardly

   Comments:

7. **Can you tell me what the complications of diabetes/cardiovascular disease are?**

   The answer indicates an understanding of the text

   ☐ Yes ☐ Nope ☐ Hardly

   Comments:

PART 2 – Perceptions

How would you qualify the following statements?

Objectives

1. **At first glance, it attracted my attention?**

☐ Strongly agree ☐ Agree ☐ Neither agree, nor disagree ☐ Disagree ☐ Strongly disagree

How could this brochure be made more attractive to you?

Comment:

2. **It held my attention**

☐ Strongly agree ☐ Agree ☐ Neither agree, nor disagree ☐ Disagree ☐ Strongly disagree

Why? How could we make this brochure more interesting for you?

Comment:

3. It is useful

☐ Strongly agree	☐ Agree	☐ Neither agree, nor disagree	☐ Disagree	☐ Strongly disagree

Why? How could this brochure be made more useful to you?

Comment:

4. I will recommend it to a friend or relative to read

☐ Strongly agree	☐ Agree	☐ Neither agree, nor disagree	☐ Disagree	☐ Strongly disagree

Could you tell me why?

Comment:

Content and topics

5. I believe what is written

☐ Strongly agree ☐ Agree ☐ Neither agree, nor disagree ☐ Disagree ☐ Strongly disagree

Is there a part that you find less believable? Could you tell me why?

Comment:

6. What it says is important

☐ Strongly agree ☐ Agree ☐ Neither agree, nor disagree ☐ Disagree ☐ Strongly disagree

Why ? Is there a specific part that you think is less important?

Comment:

7. It reminds me of some things I need to think about

☐ Strongly agree	☐ Agree	☐ Neither agree, nor disagree	☐ Disagree	☐ Strongly disagree

Could you give me some examples? If not, are there things we could add that would be more helpful to you?

Comment:

8. It gives me new ideas or leads to implement

☐ Strongly agree	☐ Agree	☐ Neither agree, nor disagree	☐ Disagree	☐ Strongly disagree

Are these things you already know? On what subject would you like us to add new ideas/news?

Comment:

9. It changes some of my thinking

☐ Strongly agree	☐ Agree	☐ Neither agree, nor disagree	☐ Disagree	☐ Strongly disagree

Why? Could you give me some examples?

Comment:

10. It could change how I do things

☐ Strongly agree	☐ Agree	☐ Neither agree, nor disagree	☐ Disagree	☐ Strongly disagree

Why? Could you give me some examples?

Comment:

Layout

11. It is easy to read

☐ Strongly agree ☐ Agree ☐ Neither agree, nor disagree ☐ Disagree ☐ Strongly disagree

Why? How could we make this brochure easier for you to read?

Comment:

12. It is easy to understand

☐ Strongly agree ☐ Agree ☐ Neither agree, nor disagree ☐ Disagree ☐ Strongly disagree

Were there any words that were difficult for you to understand? How could we make this brochure easier for you to understand?

Comment:

13. I like illustrations

☐ Strongly agree ☐ Agree ☐ Neither agree, nor disagree ☐ Disagree ☐ Strongly disagree

Why? How could we improve the illustration(s)?

Comment:

14. The illustrations are easy to understand

☐ Strongly agree ☐ Agree ☐ Neither agree, nor disagree ☐ Disagree ☐ Strongly disagree

Why? What do you find difficult to understand?

Comment:

15. I love the colors

☐ Strongly agree ☐ Agree ☐ Neither agree, nor disagree ☐ Disagree ☐ Strongly disagree

Why?

Comments:

Conclusion

16. In general, what do you think of the folder and the information it contains?

What are the positives? What are the negative points?

Comment:

17. Is there any information you would like to have that is missing from the folder?

Comment:

18. Is there anything else we haven't covered that you would like to mention?

Comment:
